# From mental health detention to health systems reform: Co-producing policy and practice recommendations with Black men, their communities, and health and social care professionals

**DOI:** 10.1371/journal.pmen.0000457

**Published:** 2025-12-15

**Authors:** Elaine Craig, Caroline Leah, Jeremy Dixon, Anna Bergqvist, Isaiah Brodrick, Debbie Best, Kim Heyes, Joy Duxbury, Alina Haines-Delmont

**Affiliations:** 1 School of Nursing and Public Health, Manchester Metropolitan University, Manchester, United Kingdom; 2 Department of Social Care and Social Work, Manchester Metropolitan University, Manchester, United Kingdom; 3 Centre for Adult Social Care Research, Cardiff University, Cardiff, United Kingdom,; 4 Research Institute of Health, University of Cumbria, Cumbria, United Kingdom; PLOS: Public Library of Science, UNITED KINGDOM OF GREAT BRITAIN AND NORTHERN IRELAND

## Abstract

“I can’t breathe,” is the phrase that has come to symbolise broad forms of systemic suffocation experienced by Black communities within mental health systems. Positioning Black men, within the context of health and social dynamics, involves understanding the unique systemic barriers they face. Using mental health detention as the entry point to explore these systemic issues, this research aimed to understand why Black men are disproportionately detained under Mental Health legislation and why they report more traumatic, coercive experiences than their White counterparts. It then co-produced solutions with Black men, their families, communities, and health and social care professionals. This is the first study to unite over 200 stakeholders to to co-produce policy and practice recommendations for change. The Silences Framework (TSF) was used as the overarching theoretical framework along with Experience-Based Co-Design (EBCD) as the participatory action research method. This paper is novel, as the application of TSF fills an important, theoretical gap in the empirical work on mental health and discrimination. Reflexive thematic analysis and methodological triangulation were used to analyse and synthesise the data including: 1:1 interviews with professionals; focus groups with Black men, family members and Black community pillars; EBCD events and parliamentary symposiums. Three themes: (1) ‘Identities Beyond the Mask’, (2) ‘Re-Humanising Detention’, and (3) ‘Radical Reform’ were co-developed with participants. Findings identify stigma, mistrust, discrimination and institutional racism are at the helm of the problem. Seven policy and practice recommendations were co-created to begin to address institutional injustice in psychiatry and better serve the Black community during mental health crisis. This paper presents an original, first of its kind study, investigating the experiences of community stakeholders and professionals and makes a significant contribution that informs policy makers and professionals working in a wide range of mental health and custodial settings around the world.

## Introduction

The words “I can’t breathe,” seared into global consciousness during the 2020 protests following the murder of George Floyd, and became a rallying cry for the Black Lives Matter movement. This phrase not only captured the literal violence inflicted by law enforcement but has since come to symbolise broader forms of systemic suffocation experienced by Black communities, including within mental health systems. Positioning Black men, particularly within the context of health and social dynamics, involves understanding the unique challenges and systemic barriers they face [1,2].

Structural racism and ethnicity-based discrimination significantly impact Black men’s health outcomes, often leading to poorer health compared to other groups [3–6]. This is compounded by social determinants such as access to healthcare, socioeconomic status, and exposure to stressors related to discrimination [7]. In addition, studies have shown that Black men, especially those who are part of the LGBTQ+ community, encounter unique forms of discrimination [8–10]. Addressing these issues requires culturally competent clinical practices and policies that recognise and mitigate the impact of structural racism without ignoring the important dynamics of intersecting identities. These intersections of identity compound the impact of discrimination on Black men’s mental health.

Institutional and structural racism in mental health care are pervasive issues that manifest through deeply ingrained biases and discriminatory practices, leading to significant disparities in access, treatment, and health outcomes for marginalised communities [11–16]. This is increasingly becoming a Global health problem due to human migration [12,17,18] with the World Health Organisation (WHO) acknowledging the profound impact of structural racism and ethnicity-based discrimination have on health outcomes [19]. These forms of racism are embedded in the policies, practices, and cultural norms of mental health institutions, resulting in unequal treatment and systemic barriers for Black individuals [18,20–23]. Racial discrimination in mental health care further exacerbates these issues [15,24].

Evidence indicates that Black men have significant care needs, but poorer mental health outcomes than White groups [25–27]. People from Black communities are more likely to experience social exclusion including racial discrimination, poor socio-economic status and unemployment, all of which are risk factors for poor mental health [28]. In addition, the mistrust of mental health care systems among Black communities is a significant barrier to seeking and receiving appropriate care [28,29]. This mistrust is rooted in historical and ongoing experiences of discrimination and abuse. Instances of mistreatment, such as the deaths of Black men while restrained or detained in mental health facilities, continue to fuel this mistrust negatively impacting help seeking behaviour [30].

During mental health crisis Black men report experiencing racist, inhuman practices that are unfair, discriminatory, and traumatic to them and their families [2,31]. These include: unnecessary and excessive use of force; being physically restrained and pinned face down on the ground; they are more likely to be tasered; diagnosed with severe mental illnesses; be put in seclusion; and less likely to be offered talking therapies [32–36]. Different routes into mental health detention also warrant further investigation [37,38].

Whilst in mental health ‘care’ Black men often face biases and stereotypes that negatively influence their diagnosis and treatment. For example, studies have shown Black patients are more likely to be misdiagnosed with severe mental illnesses such as schizophrenia, while their symptoms of mood disorders are frequently overlooked [39–41]. This misdiagnosis can lead to inappropriate and harmful treatments, further eroding the Black community’s trust in the mental health system [41–43].

In the United Kingdom (UK), efforts are being made to address these issues. The Mental Health Units (Use of Force) Act [44], was enacted to prevent the inappropriate use of force in mental health settings after Olaseni Lewis died as a voluntary inpatient in whilst being forcibly restrained by eleven police officers. This law mandates better accountability, transparency, and training to protect patients and ensure they are treated with dignity and respect [45]. Despite this, statistics from the UK National Health Service [46] show that 242.3 per 100,000 Black people were detained under UK Mental Health legislation [47], in comparison to 68.4 per 100,000 of White people. Black men are also 10 times more likely to be placed under a Community Treatment Order; a court-ordered supervision plan that allows a person with a mental illness to be treated in the community instead of in a hospital but allows a Responsible Clinician to return the person to hospital in defined circumstances [48,49]. From this picture it is clear, mental health legislation is failing to protect Black men.

To address these issues, WHO [19] advocates for the integration of human rights, equity, and intercultural approaches into public health policies, ensuring that communities subject to racial discrimination have access to comprehensive, culturally appropriate, and quality health services. Strengthening primary health care is essential for tackling health inequities driven by racial discrimination, with WHO outlining strategic and operational levers for policymakers to promote intercultural care. These initiatives aim to create a more equitable and effective health system to better serve all populations. However, there is a distinct lack of high-quality research in this area, particularly approaches that are well theorised and involve a rigorous and novel co-design approach to truly reflect the voices of the discriminated and other key stakeholders [2].

This paper presents an original, first of its kind study, that investigated the experiences of Black men, their community’s and professionals about these discriminatory practices and makes a significant contribution to inform policy makers and professionals working in a wide range of mental health and custodial settings around the world. In this paper, the phrase ‘Black men’ is employed to refer to participants’ race and includes men of Black African, Caribbean, and other Black or mixed heritage origins residing in the UK. The term ‘Black’ is capitalized to signify its role as a political identity, which acknowledges the collective experiences of anti-Black racism and the lasting effects of colonial history.

This paper applies The Silences Framework (TSF) [50] to fill an important, theoretical gap, in the empirical work on mental health, detention and discrimination Black men face whilst being detained under mental health legislation in the UK. While the study’s starting point was the disproportionate detention of Black men under the Mental Health Act, our scope expanded to include the perspectives of families, community members, charity organisations, and health and social care professionals. Detention was conceptualised as the entry point into a broader mental health care pathway, enabling us to explore systemic barriers, institutional racism, and opportunities for reform from multiple vantage points.

This was part of a bigger National Institute for Health and Care Research (NIHR) funded project (NIHR 201715) to co-produce policy and practice recommendations for change, called the ImproveAct Project. A systematic review [2] preceded an innovative co-produced qualitative study which this paper reports the findings of. This paper reports the results from the Experience-Based Co-Design (EBCD) part of the study, using the TSF [50] as an overarching theoretical framework and EBCD [51] as the participatory action method,

## Methods

**Fig 1:**
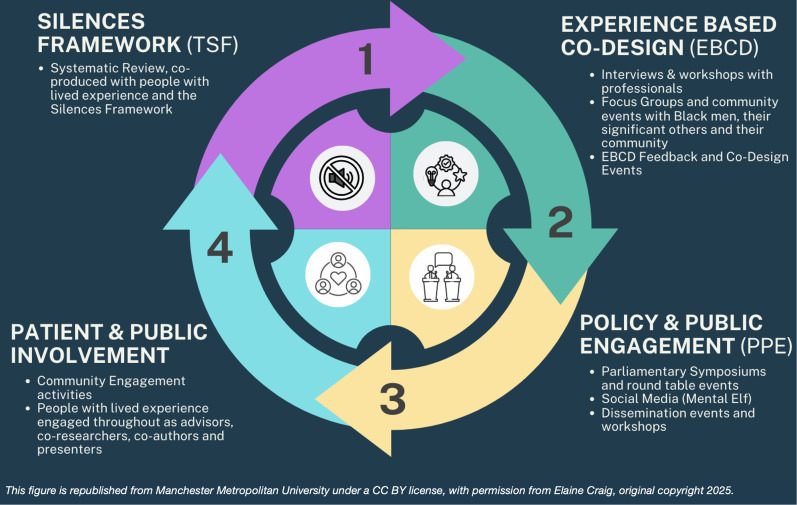
The Methodological and Theoretical Underpinning of the Stud. This circular diagram is a visual representation of how EBCD was combined with TSF, complimented by a two-part public and policy engagement strategy to co-produce a set of robust findings with rigorous theoretical and methodological underpinnings.

TSF has been designed to explore sensitive topics, that are little researched or silent from policy discourse and practice [50]. TSF begins with a review of the evidence [2] to identify ‘screaming silences’ then empirically explores and enables professionals and service users to co-design services, interventions, care pathways, in partnership. TSF was used alongside EBCD [51] to redress existing power imbalances through a collaborative, co-production focused methodology (as shown in Fig 1 below). This circular diagram is a visual representation of how EBCD was combined with TSF and complimented by a two-part public and policy engagement strategy to co-produce the robust findings for this paper with rigorous theoretical and methodological underpinnings.

EBCD typically involves individual 1:1 filmed interviews, to create collective stories. In this study, we used community and art-based collective group approaches to capture and validate experiences [52,53]. This was adapted because art-based Focus Groups (using poetry, spoken word, painting masks) offered an authentic, meaningful data collection approach to re-build generations of mistrust between the Black community, the police, academia and mental health professionals; the four main stakeholder groups within this project [54–56]. Due to the stigma attached to mental health, this approach aimed to create a supportive environment where men could share their experiences of racism, detention, and mental health crises anonymously, with the intention of minimising any fear of judgment [57].

### Data collection

Our approach was designed to engage multiple stakeholder groups, recognising that detention marks the beginning of a broader mental health care journey. Understanding systemic barriers and developing meaningful reforms required integrating the voices of Black men, their families and communities, and the professionals involved in detention and care provision.

Two data sets were collected using a purposeful sampling design [58]. The first, focused on professionals involved in the detention process, with thirteen online in-depth 1:1 interviews conducted with psychiatrists, Approved Mental Health Professionals (AMHPs), police professionals, and a clinical psychologist between February and October 2023. An EBCD Feedback Event followed this, where five interviewees validated the findings. A study leaflet, outlining the project aims and details how to be involved was emailed to professional heads of a mental health trusts and a police force, to be distributed to relevant professionals. Professionals who wanted to take part contacted the EBCD research leads if they wanted to take part. Professionals’ interviews explored what the participants role in the detention process was, why they thought Black men are detained more than White men; and what needs to change to improve Black men’s care and treatment.

The second data set explored the experiences of Black men, their significant others and the Black community capturing their experiences of mental health services, stigma, racism, and trauma. Recruitment ran from February 2023 to December 2023. Two art-based Focus Groups (FGs) were hosted in the community. FG1 had 10 x Black men and family members in October 2023. There, participants expressed their experiences through art. FG2 had 30 x participants (including 13 x Black men, and 17 x family members and community pillars) held in November 2023. There, FG1 participants showcased and spoke to their art, followed by a wider discussion about detention and mental health care. All participants were recruited from the community with leaflets circulated via barber shops, churches, and cafes.

**Fig 2:**
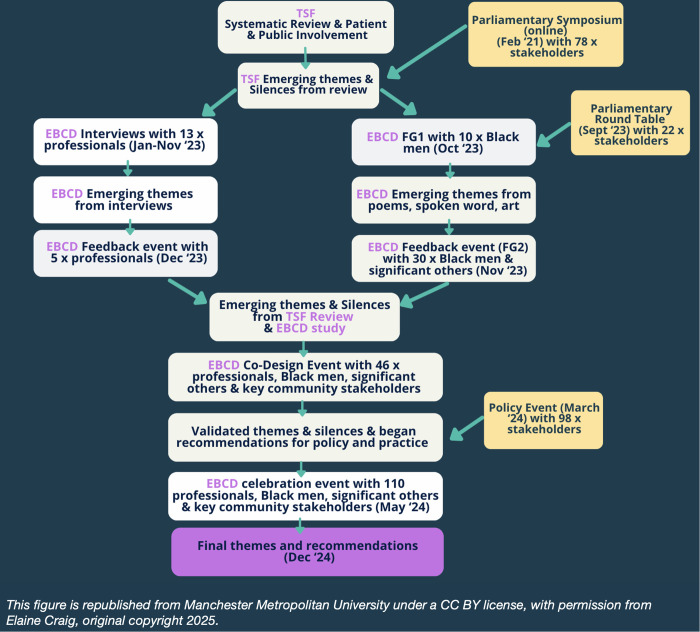
The Silences Framework and Experience Based Co-Design Data Collection Flow Chart. This flow chat visually explains how TSF and EBCD were merged in the data collection process. The white boxes represent research data of the TSF and EBCD stages. The yellow boxes represent the Public and Policy engagement events that were used to contextualise the data findings in real world settings.

Emerging findings from both data sets were presented at EBCD participant Feedback Events, refined, merged then presented and discussed again at a joint EBCD Co-Design Event in February 2024, attended by 46 x stakeholders (including Black men, family members, community pillars, members from mental health charities, professionals, and mental health advocates). A celebration event in May 2024 with 110 x attendees further contextualised the findings. This comprehensive data collection approach ensured diverse perspectives validated, then captured the wider understanding regarding mental health system failures and ways to improve practice. These multiple stages can be visualised through Fig 2, below. This figure is a flow chart visually explaining how TSF data and EBCD data were collected and merged for into the final results. The white boxes represent the TSF and EBCD data collection stages. The yellow boxes represent the Public and Policy engagement events that were used to contextualise the data findings in real world settings. The ‘Results’ section of this paper reports the final themes and recommendations co-produced by all participants.

### Ethics

The study was approved by Health Research Authority and the Health and Care Research Wales (IRAS ID 310503, REC Reference: 22/HRA/2684). All participation was subject to informed consent. Participant information packs were given to all participants identifying risks and benefits of participation, the consent process, how distress would be managed, and the secure data storage procedures. Individual written consent was gained for the professionals prior to the interviews. Their data was pseudo-anonymised, and each participant was given a unique identifier (e.g., Professional Interview, AMHP).

Verbal group consent was obtained on the day for both the FGs, and their data was pseudo-anonymised with group identifiers (e.g., *FG1, Black man* or *FG2, carer*) to re-build trust and ensure anonymity. Informed consent for EBCD event participants was obtained twice: written at the point of registration and verbal at the start of the event. Their views were pseudo-anonymised with group identifiers (e.g., *Professional participant at EBCD co-deign event*). Event attendees who did not want their views captured wore rainbow lanyards to indicate this.

Due to the highly sensitive and identifiable nature of the data, including art-based and filmed spoken-word outputs, and disclosures of witnessed racism and use of force by professionals, the full dataset cannot be publicly shared. Data may be available from the corresponding author on reasonable request and subject to appropriate ethical review.

### Epistemic tensions and negotiation in co-design

During the joint EBCD Co-Design Event, epistemic tensions between professionals and Black men (particularly around divergent understandings of “risk” and “aggression” discussed the Results section) were intentionally surfaced and explored. Professionals interpreted aggression through clinical or procedural lenses, shaped by safety protocols and institutional norms. However, the data suggested these interpretations were also influenced by racialised stereotypes, particularly the trope of the ‘big Black dangerous men’.

In contrast, Black men, families and their communities described aggression presentations as a ‘fight or flight’ trauma response rooted in systemic fear, historical injustice, and lived experiences of racialised harm including the fear of death. These differing epistemologies created potential friction in co-design discussions, which the research team anticipated and carefully facilitated through storytelling, art-based data, and structured dialogue. This enabled professionals to re-frame aggression not as a threat, but as a legitimate expression of distress.

To ethically support this, and other emotionally complex conversations, the research team implemented a series of safeguards: event chairs from the outset established ground rules of respect and listening in the room; a mental health nurse was present to manage distress; a breakout ‘breathing room’ was available; and culturally trained independent advocacy was offered to participants with lived experience. These facilitative strategies were not only ethical but also procedural enactments of the study’s theoretical commitment to addressing epistemic injustice. By creating space for Black men’s knowledge to be heard and validated, the co-design process actively disrupted epistemic asymmetry (the imbalance in credibility and authority between professionals and participants) and positioned Black men’s lived experience and the community’s trauma of systemic racism as central to shaping mental health reform.

### Public and policy engagement (PPE)

EBCD [51] allowed us to listen to the silences of the Black men and their communities. Additionally, central to TSF is *‘Working With the Silences’* where researchers fully contextualise and integrate these silences into real world practices [50,59]. To integrate the silences found, the following PPE events were held:

- Online Parliamentary Symposium (February 2021) with 78 x stakeholders
- Black community mental health celebration (May 2023) with 48 x stakeholders
- Police Professionals workshop (August 2023) with 12 x stakeholders
- Policy Roundtable Event (Sept 2023) with 22 x stakeholders
- Parliamentary Symposium (March 2024) with 98 x stakeholders

At all these events Black men and their significant others co-presented the findings to government officials and civil servants supported by the research team. Through these, we co-developed the study’s ‘Policy and Practice Recommendations’.

### Analysis

All data was thematically analysed using Braun and Clarke’s 6 phases of reflective thematic analysis [60]. Two experienced researchers analysed the professionals’ interviews (CL, JD). After analyzing 5 transcripts 122 codes, with 110 overlapping were identified. These were discussed, and a coding framework was agreed and circulated to the wider research team for feedback. Subsequent transcripts were then coded using the agreed framework, with both researchers keeping note of any new emerging themes to discuss in future meetings. Codes were collated into 5 overarching themes: **1. ‘*Stigma, racism and discrimination****,’* **2.** ***‘Risk and fear’***, **3.** ***‘Social cultural and political factors****’,* **4.** ***‘Mental health service provision’****,* **5.** ***‘Assessment and intervention*’** with 5 subthemes. These were presented and triangulated with the professional participants at an EBCD feedback event online, where the final themes and codes were agreed prior to being merged with the data set two analysis.

Two experienced researchers and one junior researcher analysed the FG data (AHD, EC, IB) including masks, poetry, stories, and spoken word. This was conducted using a combined approach of Thematic Analysis [60] and Critical Visual Methodologies. This dual approach allowed for a comprehensive examination of the poetic, narrative and visual dimensions of the artistic outputs. Thematic Analysis provided a systematic framework for identifying, coding, and interpreting patterns across the textual and visual data, facilitating the organisation of diverse expressions into meaningful themes. Concurrently, Critical Visual Methodologies were employed to interrogate the masks and how the artworks represented and communicated complex issues of racism, identity, and power. This lens enabled a critical exploration of the symbolic and contextual significance embedded within the art, emphasising the sociopolitical dimensions of participants’ experiences.

This yielded a total of 79 codes that were independently identified and after a discussion with the analysis team, were collated under 11 overarching themes. The 11 themes were discussed with the lived experience co-researchers, PPI group members and the chief investigator (AH) then re-defined, re-named and clustered into five overlapping themes: 1. ‘***Knowing me, not just the Black man’****,* 2. ‘***My community is my support’***, 3. ‘***My treatment knows no care*’**, 4. ‘***Mistrustful medication’****,* and 5. ‘***Race inside mental health power structures***’ with seven sub-themes.

The unification of both data sets is essential to meet the four core EBCD principles: ‘immersive’ and ‘emic understanding’, ‘empathy’, and ‘reflexivity’ however EBCD studies are critiqued for not adequately reporting how the data was synthesised and analysed [61]. Methodological triangulation was employed to consolidate all findings from the TSF Review [2], the professional’s interviews, and the Black FGs [62,63]. The five overarching and five sub-themes from the professional’s data were analysed next to the five overarching themes and seven sub themes from the FG data following Meijer et al. [64] three triangulation approaches: intuitive, procedural, and intersubjective. All three approaches were applied to merge the data sets collating and comparing all findings using Padlet software.

Researchers noted (1) when, where and how the FG data and professionals spoke to one another, (2) where they disagreed, and (3) where they posed different perspectives on the same challenges and therefore gave richer, deeper insight. This resulted in the three ordinate themes and nine subordinate themes reported in the ‘***Results***’ section of this paper. These themes were co-presented, triangulated and validated at the EBCD joint event. The themes spoke to the problems of mental health care that Black men in crisis experience but they did not yet offer any solutions to the problems. Solutions were discussed at the EBCD joint event where the ‘***Policy and practice recommendations***’ suggestions began.

During the merged data analysis, the researchers also noted where there was evidence that the two participant groups misunderstood each other or lacked knowledge in an area. These nuances were used as discussions points at the joint EBCD events. Hence, the final themes: 1. ‘***Identities beyond the mask’*** 2. *‘****Re-humanising detention’*** and 3. ***‘Radical reform’*** were interpreted through the EBCD event discussion and PPE events.

At these events’ researcher notes, illustrated minutes, and post-it notes recorded the interpretations of the themes important to these discussions, whilst feedback forms allowed attendees to provide anonymous insights. From these, 75 suggestions to improve policy and practice were identified. Duplications were removed, and the remaining considerations were cross-checked with the existing recommendations in the Mental Health Act Reform White paper [65]. Solutions already in the White paper were removed leaving 7 final considerations. These considerations were validated by the wider research team and lived experience PPE group then co-presented at the Parliamentary Symposium with 98 stakeholders. Our final ‘***Policy and practice recommendations***’ focus on tackling systemic racism and preventing mental health crisis prior to detention.

### Rigour

In recognition that the themes are not inherent in the art itself but are constructed through the researcher’s critical analysis process [66] researchers own interpretations of race and racism, including biases and limitations had to be addressed for rigour. Five researchers analysing the data were White and one was Black. To build rigour, the team intentionally set measures to assess for Whiteness in the analysis and interpretation.

‘Whiteness’ refers to the phenomenon where the perspectives or assumptions of White individuals are intrinsic within the analysis and interpretation of the data [67–69]. This can potentially lead to inaccurate conclusions that overlook or minimise Black people’s reality essentially treating White experiences as the universal standard. The research team hosted an anti-racism workshop to reflect on Whiteness in academia, their own positionality and how that could influence the data. This learning was continually revisited and reflected upon on during all project meetings. All emerging findings were discussed, revised, amended, named and validated with Black members of the project’s lived experience group as well as the PI and the wider research team.

Casey and Murphy’s [70] four evaluative criteria - truth value, applicability, consistency, and neutrality - were applied. ‘Truth’ was achieved through participant quotations that objectively captured experiences [71]. ‘Neutrality’ and ‘applicability’ were achieved by reaching data saturation, with new data not introducing additional themes. ‘Consistency’ was ensured by co-presenting and validating results to over 200 stakeholders at multiple EBCD and PPE events.

## Results

While the study began with Black men’s detention experiences as the focal point, the thematic analysis integrates perspectives their families and communities, mental health charities and advocates, and professionals. These perspectives reveal detention as an entry into a wider system characterised by systemic inequities, and they underpin co-produced recommendations for change.

**Fig 3:**
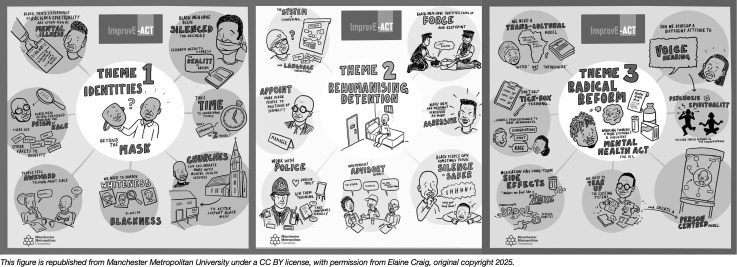
ImproveAct Theme Illustrations. To make the ImproveAct findings accessible, the themes were visually organised into one infographic illustrations that capture discussion of the themes within the context of the PPE events. This is a set of three themes places next to one another in sequence with illustrations of the findings.

The emerging themes were organised into three ordinate themes with nine subordinate themes. The themes prioritise the voices of Black men and their community. Themes were visually organised into infographic illustrations to capture discussions from the PPE events and can be seen in Fig 3, below. These themes identify the problem of inhumane detention, contextualised through the EBCD event discussions to capture the broader socio-economic mix of gendered and racialised experiences. These include social stigma, institutional coercion, and the Black communities’ perceptions of services.

### Theme 1. Identities beyond the mask

This theme reflects participants’ calls to be recognised as whole individuals rather than reduced to racialised stereotypes. The “mask” symbolises the imposed identity of “Black man” as seen through the lens of bias and institutional racism, obscuring personal histories, trauma responses, spirituality, and community connections. Participants described wanting to remove this mask so that professionals could see their humanity, complexity, and individuality. This framing sets the context for their experiences and the quotations that follow.

Black men from FG1 and the EBCD events felt like being Black was the first and sometimes only way that professional looked at them. This theme describes their request to be seen beyond the monolithic mask of race and be understood through their intersecting identities, including the lenses of spirituality, stigma, and the trauma of racism.

#### 1.1. Identity.

As one participant explained, professionals often failed to see the person beyond the stereotype of the “big Black man,” reinforcing the sense of being masked and misrepresented.

*“Each case is different and seeing each person as an individual is important. We are constantly evolving. It’s essential to ask someone in crisis about their lived experiences. Having a mental health issue is a journey in a difficult and racist world. If external factors make the person sick, then racism can seem like an illness and have lasting effects. The culture around mental health must change for us all to be well.” (FG2, Carer of detained Black man).*

Black men and health and social care professionals agreed that practises should adopt person-centred approaches that acknowledge the traumatic weight of racism in shaping identities. They felt that reforms must address the tendency to treat Black men monolithically in both law enforcement and mental health services, emphasising the importance of being treated as individuals rather than inappropriate Black stereotypes.

Some EBCD event participants highlighted that care which only acknowledges one aspect of an individual (e.g., race), fails to provide true person-centred care. The EBCD discussion on this, expanded this point by describing how skin tone, gender, sexuality, class, region all plays a part in how people exist in the world and can dictate how people are treated. This means both the individual and the community are equally important.

FG1 and FG2 highlighted what community means to Black people in terms of support and healing and how they felt that differs from the predominately individualist culture of White communities. An example was given by a detained Black man where there was not a visiting room big enough in the hospital for his family to visit. Professionals at the events agreed that community-based support is a vital part of mental health services, particularly to reduce mental health crisis, and was referenced many times when Black men and Black communities shared examples of good practice. Community initiatives that are led, specifically by community pillars, but more broadly for and by people with lived experience need piloting, endorsing, and long-term funding.

#### 1.2. Racism.

FG1 and FG2 participants shared their experiences of racism, expressing their opinions that some of the harm faced by people in mental health services is due to experiences of racism being treated as a part of their mental illness. The EBCD event discussions unpacked how past experiences of racism and anti-Blackness could be intrinsically linked to fear of services and being detained. Clinical professionals at the EBCD event added how a traumatic fear response, caused by historic trauma, could possibly present as a fight or flight reaction that could be misinterpreted as aggression:

*“…a need for teaching/training about aggression not being aggression but as a justified result of trauma and fear of death” (FG1, Black man).*

All the study’s participants pointed out that there is a stigma surrounding Black men’s mental health that is often associated with negative perceptions of aggression and stereotypes of ‘big Black men’. A solution discussed to combat this was that it is crucial to examine how Whiteness influences the provision of mental health services, with a focus on ensuring services are culturally sensitive and person led. This would help practitioners recognise when an individual’s behaviour is a trauma response rooted in their racial discrimination.

#### 1.3. Spirituality.

Findings from FG1 and FG2 highlighted that religion and spirituality form important parts of many Black men’s identities. They expressed, that churches and faith communities were often reported as central hubs within their communities and is where some of the FG1 participants said they turn to for support or peace during difficulties and crisis. FG2 discussions included how religion and spirituality should be integrated into mental health support for Black communities.

*“Meaningful change will only happen if we work collaboratively with the Black community including faith leaders” (FG2, church minister).*

Black men knew that diagnostic manuals consider an include spiritual and religious aspects as a dimension within diagnostic categories (including schizophrenia and other psychotic disorders, depression, substance use disorders, anxiety, PTSD, and personality disorders). Therefore, they said, they are often hesitant to express any spiritual beliefs/practices for fear these will be pathologized. This demonstrates:

*“…the need for research and training of spirituality in Mental Health services so that spiritual beliefs/practices are honoured and embedded as support and not misdiagnosed as psychotic symptoms” (FG2, carer to detained Black man)*.

Professionals in the EBCD events acknowledged this but highlighted that more research was required to better understand this considering mental health assessments. A solution discussed by all was that there is a need to integrate religious/spiritual practices into mental health care in a non-pathological way. This involves creating culturally sensitive approaches to mental health assessment that validate and incorporate diverse spiritual beliefs and rituals. Exploring whether faith leaders could be involved in mental health assessments when spiritual practices were disclosed was discussed but warranted further investigation.

### Theme 2. Re-humanising detention

This theme focuses on restoring dignity, compassion, and understanding to the detention process, which many participants felt was de-humanising and disempowering. “Re-humanising” refers to ensuring individuals in crisis are treated as people first, not as dangerous risks or problems to be managed. Black men, their families, and professionals highlighted how clearer communication, cultural awareness, and empathy could transform these moments into opportunities for care rather than trauma. The quotes in this section illustrate the urgent need for such change.

#### 2.1. Power imbalances.

FG1 and FG2 participants felt that the current detention process lacks transparency and humanisation, leaving individuals feeling powerless and disconnected from their care. They described being “*treated”* not “*cared for”.* This highlighted that policies must prioritise humanising communication before, during and after detention, ensuring individuals understand what is happening to them and why. Demystifying jargon and making pathways to care more visible and accessible were specifically highlighted by these groups:

*“Information needs to be provided to patient and their loved ones about what is happening to them and their rights. That information needs to be in multiple accessible forms including QR codes to a film. Not just in written formats” (FG2, carer to detained Black man).*

FG1 and FG2 participants felt it was essential to address the arbitrary nature of the detention process, which they felt, often disregards the individual’s needs and rights. Families in FG2 said they worry about making complaints in case it worsens their loved one’s treatment fearful that complaining may: “put a target on their back”.

Families elaborated that these fears ran so deep they when their loved one was discharged if they feel unwell again families would actively ‘hide’ their loved one fearing and anticipating further re-traumatisation should the need detaining, from the services supposed to protect and heal them.

This was discussed at the EBCD event and professionals involved in the detention pathway agreed that to empower individuals, policies should encourage open communication without fear of repercussions allowing families and carers to voice concerns openly.

*“Mental Health professionals need to take time to reflect with patients, carers and staff who have been involved in the Mental Health Act if they have been heard. Do not judge the voice of carers. Let them speak and listen. Involve them in care” (Professional participant at EBCD co-deign event).*

#### 2.2. Misconceptions.

Black male participants in FG1 and FG2 contextualised expressed that their fight or flight response to being detained stems from a place of fear of death and/or dying in police custody. This fear, they feel is legitimate, not imagined, and stems from the multiple deaths of Black men reported in the news. When discussed at the EBCD event it was highlighted, by a senior police professional, that this fear could be misinterpreted by police responding to a mental health crisis as aggression. They expanded police do not have mental health presentation training and a solution was discussed saying that policymakers must prioritise understanding aggression and risk from both the perspectives of professionals and individuals with lived experiences to dispel these misconceptions:

*“…there’s a perception of Black men generally in society but also in mental health services as well, that they are generally more aggressive, they’re described as aggressive more than not, and I think a willingness to understand where that behaviour comes from and what’s driving that behaviour is clouded by that view” (Professional interview)*

Professionals at the EBCD and PPE events found this learning insightful and highlighted a need to explore the nuances of risk perception by questioning who is being protected from what risks during detention then creating training to explain these nuances:

*“We need a resource to train all practitioners about what we hear today [the three themes] Black men’s experiences of detention, the police, the inequalities faced and the harm as a result of this” (Police Professional at EBCD co-deign event).*

#### 2.3. Injustices.

A finding from all data sets was that it was crucial to recognise that the act of detention under mental health law can itself perpetuate a cycle of violence and aggression. Black men in FG1 and FG2 said that is particularly true for Black men who have experienced this as a form of victimisation:

*“When they’re unwell and they don’t think they need admission, but the police turn up and they think, last time, this is what the police did to me. I need to run, I need to get out of here, it’s going to increase their fight or flight” (Professional participant at EBCD co-deign event).*

FG2 participants at the EBCD event, explained that a person who exhibits aggression during detention may be doing so to protect themselves from violence that they are experiencing, or that fear they may suffer, during detention. They went onto describe that the perpetuated feelings of injustice Black men and their communities experience, can result in them rejecting mental health services and relying on support within their own community. FG1 participants expressed the impact of this means they often struggle to re-discover their voice after detention and often feel that suffering in silence is safer:

*“There’s a cage on the mouth…they caged up our mouths…and were unable to speak our hearts and what’s in our minds” (FG1, Black man).*

Due to experiencing these injustices FG1 participants shared they often do whatever they need to do to get out of mental health care and leave services carrying more trauma than when they entered. When sharing this in FG2, their significant others and the community spoke of the collective community trauma that this injustice in psychiatry has upon the whole community. Collectively event attendees identified that reforms needed to be radical to elicit the system changes needed in the mental health care. These are included in the Policy and Practice Recommendations below.

### Theme 3. Radical reform

This theme captures participants’ belief that incremental changes are not enough. The mental health system requires bold, top-down and bottom-up co-designed systemic transformation. “Radical reform” means re-thinking detention, assessments, treatment, and medication practices to dismantle institutional racism and embed culturally responsive, person-centred care designed with intersectionality in mind. The experiences shared here highlight how deeply ingrained inequalities are, and how policy, practice, and training must shift to address them.

*“How can we ‘fix’ people in a broken system, one which suffers from institutional racism? We need to consider conscious and unconscious bias in professionals, challenging racism in practice. For practice to be truly progressive and based on human rights, this includes reporting and logging incidents of racism.” (FG1, Black man).*

The quotations illustrate specific areas where participants see opportunities for fundamental change.

All participant’s agreed current legislation does not protect Black men and that there is a need to work together towards a more equitable and inclusive Mental Health Act for all by reforming mental health care, treatment, and medication. Participants identified key domains for reform, beginning with transforming care provision.

#### 3.1. Care.

Transforming mental health care for equitable and culturally sensitive support was a unanimous priority for all participants in all data sets. They felt the UK’s mental health care system should be characterised by racism-informed, person-centred, needs-led and trauma-informed approaches:

*“A more person-centred admission plan...I think maybe more support around the person having their voice heard...Basically, it being more person centred” (Professional interview).*

Ideas on how this could happen included having opportunities for carers and significant others to have joint decision-making with practitioners instead of being forced into pre-existing care packages:

*“Maybe afterwards, the care coordinator or someone to speak with the person to get their feedback of how the detention process was for them...it’s the person in the middle of it and getting their feedback is probably the most important thing in how we change legislation and make a positive change” (Professional interview).*

FG2 highlighted that they wanted mental health care pathways to encompass community-based care, from pre-detention through to post-discharge, with a focus on addressing trauma experiences during detention. All participants agreed involving families in care decisions and explaining mental health legislation are crucial steps in fostering understanding and support.

Diversifying the healthcare workforce was discussed at the EBCD events, as means to tackling this however professionals highlighted that, this is *not* always the solution. Incorporating de-racialised psychiatry principles into assessments and interventions (e.g., spiritual beliefs and practices) were highlighted at the EBCD events as essential for providing culturally adapted care and a step towards tackling institutional racism. So too was the need for professionals’ supervision to have a reflective practice component that re-visits ‘Whiteness’ in their practice. Reforming treatment practices was another critical focus.

#### 3.2. Treatment.

FG1 participants expressed that they believed professionals lacked understanding of cultural beliefs and again, spiritual practices were given as an example when assessing for psychosis and schizophrenia. FG1 and FG2 highlighted the need for: *“…culturally appropriate education around mental health difficulties and how those can manifest” (FG1, Black man).* This gap in knowledge can lead to unintentional racist practices from professionals around diagnosis and medication management (e.g., diagnosis is applied via a Eurocentric diagnostic manual).

Interviewed professionals raised the need for treatment reforms that require practitioners to put the person before the practice. One professional participant from the co-design event asked, *“can we have these themes framed and taught about in light of MH assessment so practitioners can have them as a culturally appropriate checklist against implicit racist decision making” (Clinical Psychologist at EBCD co-design event).*

At the EBCD event it was discussed that the radical-ness of this means instead of operating on the principle of what is the ‘least restrictive approach’, practitioners’ default should be ‘***what is the most supportive approach***’ for this person. Medication practices also emerged as an area demanding fundamental change.

#### 3.3. Medication.

FG1 and Black men and carers in FG2 expressed mistrustful concerns regarding anti-psychotic medication and the medical claims given about how it can provide relief from hearing voices. They wanted health system reforms to address the systemic issues negatively impacting the Black community’s trust in healthcare specifically regarding the over-medication of Black men, medication side effects, and concerns about control:

*“There is a legitimate suspicion of how medications are developed, what pharmaceutical companies’ real aims are and about the safety of medications… medications are generally developed and tested on White populations… If you thought about the long-term, from the early stage and either got the dose right or minimised side effects to make it more tolerable…then would almost certainly reduce the chance of …re-admission” (Professional interview).*

The EBCD event discussion highlighted there is a belief in the Black community that drug testing primarily caters to White populations. They explained this contributes to the mistrust the Black community has with the healthcare system giving examples of over medicating, mores severe side effects of medication and ineffective treatment of Black men.

Whilst no concreate solutions were offered at the EBCD events regarding this, it was acknowledged that historically Black men were underrepresented in drug trial though this is no longer the case [39]. It was also highlighted that engaging the Black community in the growing field of Neuropharmacology [72,73] was discussed as a step towards re-building the broken trust.

### Policy and practice considerations

This study aimed to unite various stakeholders to identify key actions for change. During our EBCD and PPE events, attendees emphasised that achieving “real change” requires bold, re-imagining of current services. Over 200 people (including Black men, their significant others, health and social care professionals, third sector advocate agencies, mental health charities, policy and governmental ministers, and the Black community) helped us to validate and re-frame the emerging policy and practice recommendations. Given the historical under-representation and silencing of this group in research, it was crucial to report their considerations authentically. Future research should focus on the feasibility, piloting, and comparison of these recommendations, which were beyond this study’s scope

#### 1. Establish a community-led advisory group to address detention led inequalities.

*“Let’s set up an independent advisory group […] once every three months to challenge mental health” (Police Professional at EBCD Co-Design Event)*

Stakeholders proposed forming a community-led independent advisory group to address inequalities and racism in mental health detention. This group would include faith leaders, community pillars, individuals with lived experience, social workers, advocates, police, NHS mental health professionals, and local authority representatives. Each member would lead actions in their area and nominate workplace champions to implement changes.

The group would investigate deaths of Black people detained under the mental health legislation particularly those following restraint and learn from historic race-related deaths. Following the UN’s call to end impunity for human rights violations, the group could set up an equivalent system to Domestic Homicide Reviews to investigate the circumstances of a death during detention using the lens of racism and mental health inequalities.

Meeting should be held quarterly along with an annual public meeting, where the group would gather community feedback, address issues, and ensure accountability. This approach aims to rebuild trust and address the power imbalance between the community and mental health institutions, creating a more equitable and just mental health system.

#### 2. Develop ‘Go to Mental Health Hubs’ in the community.

*“Community based support is key – best space to open up, un-silence silences, re-build trust in oneself, the community and support services...” (FG1, Black Man)*

Stakeholders proposed creating community-based ‘Go to Mental Health Hubs’ to shift from a medicalized model to a community-oriented, safe space. These hubs would foster mental health conversations before crises, serve as first contact points for referrals, and provide training on handling crises and safeguarding. Suggested locations include churches, community cafés, and centres. The hubs would host advisory group meetings and feature ‘living libraries’ showcasing recovery stories of Black men. They should offer 24/7 access to information on the detention process and rights, using multiple formats. Implementing these hubs would create supportive spaces for proactive and inclusive mental health care.

It is crucial that patients and their chosen support networks are informed about the detention process and their rights in multiple accessible formats, including QR codes linking to short films, not just written materials. The ‘Go to Mental Health Hubs’ should offer 24/7 quick and confidential access to this information, along with signposting to available support and further guidance on next steps. This information should cover assessment, hospitalisation, tranquilisation, medication, and side effects.

#### 3. Enable advocacy, rights and complaints in mental health trusts.

*“I have supported my son through local, medium, high and low secure forensic services for over fourteen years.. the hardest part has been challenging practices inequalities, disparities, gaslighting and coercion to name a few within services.” (FG2, carer of detained Black man)*

To create a fairer mental health system, it’s essential to establish culturally appropriate, independent advocacy within Mental Health Trusts. This advocacy empowers patients to challenge assessments, support, and medication decisions, promoting fairness and transparency. Complaints about perceived injustice or racism must be thoroughly investigated, with appropriate penalties for responsible staff and patients informed of outcomes. These measures will address the culture of racism and rebuild trust between the Black community and mental health institutions, fostering accountability and respect for all individuals’ rights.

#### 4. Rebuild trust between the police and the Black community.

*“I think there’s a perception of Black men generally in society but also in mental health services as well, that they are generally more aggressive, they’re described as aggressive more than not, and I think a willingness to understand where that behaviour comes from and what’s driving that behaviour is clouded by that view” (Professional Participant).*

To rebuild trust between the police and the Black community, it’s essential to address the perception of Black men as inherently aggressive. Enhanced police and mental health training should include co-developed short films featuring Black men’s experiences to combat stigma and provide a nuanced understanding. Training should also help officers recognise PTSD signs and understand that challenging behaviour may stem from past negative experiences. Community Hubs can facilitate open conversations between the police and Black communities to understand each other’s roles and avoid conflict. Implementing these measures will foster a more respectful and understanding relationship.

#### 5. Shared decision making and person-centred care.

*“They were describing the framework solely from their professional perspective and trying to tell us, the patient, carer, how we should use it. That’s not equity, co-production, or partnership working. My request is simple. It’s essential we work it out together, sharing learning and understanding” (FG2, carer of detained Black man).*

To foster equity and partnership in mental health care, it’s essential to involve patients and their significant others in meaningful ways. Black men and their community’s emphasise the need for collaborative care decisions, seeing individuals beyond their race and involving chosen advocates like faith leaders. Professionals must listen to patients, carers, and staff to ensure all voices are heard. Family intervention and cultural elements are crucial, with creative and non-pharmaceutical treatment options available. A culturally appropriate Buddy system can address loneliness in inpatient settings, extending support to family members during and after detention.

#### 6. Reform the mental health assessment process.

*“Start with the people. Listen to who they are, what they need, to develop a care plan for them, do not try to fit them in already existing care packages” (Black man at EBCD event).*

To create a more equitable and effective mental health assessment process, it’s crucial to start with the people, tailoring assessments to individual needs. Reforming assessment tools involves developing culturally appropriate methods with input from Black communities, researchers, and practitioners. Allowing more time for assessments, such as a minimum of two weeks, ensures thorough evaluations.

It is important to include intersectional identities in assessment and treatment and consider how this informs tailored support whilst keeping anti-racist, anti-stigmatic practices at the forefront. Individuals and their carers should have equal input in decision-making about detention and treatment, with strong justifications provided to all parties. Implementing these recommendations will create a more inclusive and person-centred assessment process that respects and empowers individuals and their communities.

#### 7. Reforming medication practices in mental health care.

*“Having been in the mental health system and now medication free, I see that practice ideas and the medical model is out-dated and broken…was told I would be on medication for life, but I am not and won’t be. Medication isn’t always the answer, communication and talking therapies are important. However, communication with people outside your own race can be difficult” (Black man at EBCD event).*

To improve mental health care, it’s crucial to develop a transcultural system for administering psychiatric medication that considers physiological, metabolic, and psychological differences between races. This system should include access to an independent, culturally trained pharmacist and an advocate for patients to ensure comprehensive and culturally sensitive support.

Medication should be reviewed if a psycho-social intervention is chosen to avoid unnecessary side effects. Non-pharmaceutical interventions, like communication and talking therapies, should be prioritized, especially for Black men. Implementing these recommendations will create a more inclusive and effective mental health care system that respects diverse needs and promotes holistic well-being.

## Discussion

In this study detention was conceptualised as the starting point of a mental health care journey that exposes structural and institutional racism, with our analysis integrating multiple perspectives to co-produce actionable policy and practice recommendations. Hence the findings lead with experience of Black men (characterised by systemic inequities), then contextualised these experiences by integrating multiple perspectives to co-produce actionable policy and practice recommendations.

This work is framed through an intersectional lens [74,75] that recognises how Black men’s mental health experiences are shaped by multiple, overlapping social identities (including spirituality, sexuality, class, and intra-community differences) that compound stigma and discrimination in complex ways. Intersectionality enables a deeper understanding of how these identities intersect to affect Black men’s encounters with mental health systems, informing tailored approaches to care.

The originality and significance of this paper is that it is the first study to unite over 200 stakeholders to centre the lived experience of Black men and co-produce policy and practice recommendations using a strengths-based approach [76] informed by shared decision-making [77] and relational public service management theory [78,79] and EBDC [51,80].

Our focus was to co-create solutions that prioritise peoples lived experience to identify and create healthy systems with citizens and stakeholders as partners. Broad goals and missions to reduce racism in mental health give public health systems a strategic focus in improving the experiences of Black men detained under the Mental Health Act. However growing research shows that complex systems cannot be tightly controlled [73].

**Fig 4:**
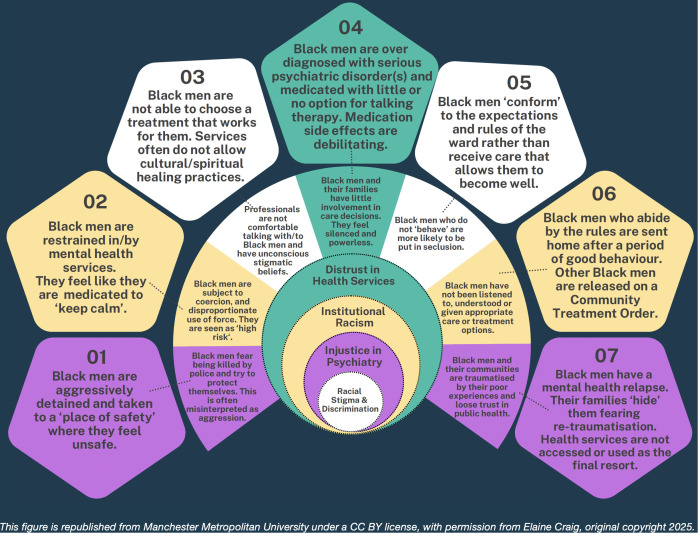
Centring Black Men’s Mental Health Experiences within the Wider Systemic Issues of Systems Harm. This semi-circular diagram is a visual representation that centres the Black Men’s Mental Health Experiences of detention within the wider systemic issues of systems harm. It signifies that improving pathways to detention alone will not be suffice without dealing with the wider roots of societal harm perpetuating cycles of trauma.

To produce better mental health outcomes; learning from stakeholders with negative life experiences of racism in this domain and wider societal ecologies is the harder complexity task to improving outcomes. Fig 4, below, outlines these complexities by centring the reflective experiences, perceptions and beliefs of the Black men and their community embedded within the societal context of racial stigma and discrimination, injustice in psychiatry, institutional racism and distrust in health services.

This figure is a visual representation that centres the Black Men’s mental health experiences of detention in the outer sphere, the knock-on impact this has on their families and communities in the second sphere, set within the multi systemic issues of social harm in the four centre spheres. It signifies that improving pathways to detention alone will not be suffice without dealing with the root causes of systems harm perpetuating cycles of racism, stigma, discrimination and injustice.

Due to its complexity, public health service requires ongoing learning beyond the broad goals and strategic mission to enable this adaptation to produce better value outcomes. As Smith [78] highlighted we should shift our focus away from merely enhancing services. When services are the primary focus, the outcome remains just services. What people truly desire is support, meaningful relationships, practical assistance, and to feel understood. Complexity and wider societal ecologies of in health care therefore invite re-examination and an openness to flexibility and experimentation in service delivery to identify barriers to co-creative solutions that interacts with patient’s lived experiences of institutional racism in engaging with the health service informed by shared decision-making [69,77].

Central to this dialogue is the concept of epistemic injustice [80–82], which occurs when Black men’s knowledge and lived experience are systematically undervalued or dismissed within mental health settings, resulting in epistemic asymmetry and power imbalances between professionals and service users. This injustice manifests as institutional ignorance and silencing of Black men’s voices, undermining trust and engagement. By foregrounding epistemic injustice, we provide a lens through which to analyse the tensions and distrust observed between participants and professionals, highlighting the need to rebalance power dynamics through meaningful recognition of lived expertise.

Institutional racism and structural racism are related concepts that present barriers to co-create solutions that prioritise peoples lived experience of this kind in mental health services, but they focus on different aspects of how racism is embedded in society. Institutional racism refers to the systemic inequalities and discriminatory practices within psychiatric institutions that negatively impact Black communities [83]. Structural racism is a broader concept that refers to the systemic and institutional practices, policies, and norms that create and perpetuate racial inequalities in various aspects of society [84].

Findings suggest that structural racism (including Whiteness, implicit bias, and negative Black stereotyping) exists within the mental health system and is perpetuating a cycle of institutional injustice in psychiatry for Black men and their community [6,12,14]. Implementing and enforcing anti-discrimination laws, and affirmative action within practice guidelines and creating data collection templates and accountability to collect and analyse data on racial disparities to inform policy decisions and hold institutions accountable for progress towards equity are identified as priorities for change [84–86].

Despite the availability of techniques to address structural racism, there is a lack of sustained commitment to implementing these strategies in psychiatric practice [86,87]. Adopting and implementing the policy recommendations from this study are crucial for achieving, maintaining and sustaining long-term change. Public value in the context of mental health is process of co-creation and shared decision-making where persons with lived experience of mental ill health and services engage in a process dynamic relationship and dialogue to restore quality of life through the interactive partnership [80]. This holds true for public services more generally; value is created at the nexus of interaction [88].

Epistemic injustice [81] occurs when individuals’ knowledge and experiences are undervalued or ignored as credible in this dialogue because their capacity as knowers and expert witnesses lack power in the dynamic interaction between the epistemic stakeholders in question. This injustice can manifest as institutional ignorance and the silencing of experiences [82]. Despite some practitioners validating the needs of individuals [89], addressing epistemic injustice requires open dialogue and valuing lived experiences in mental health practice, policy and research [81,89]. Suggestions how to re-balance the power as part of the reforms are in the study recommendations.

The intertwined negative stigma of mental health and the negative stigma of Black men being ‘big bad and dangerous’ has permeated the professionals’ boundary of the mental health system [90,91]. Stigma significantly exacerbates structural racism and institutional injustice in psychiatry in several ways [92]. Stigma around mental health can prevent individuals from seeking help. This is particularly pronounced for Black men where mental health issues are stigmatised, there is a permeant narrative whereby Black men fear for their life whilst during detention, leading to an underutilisation of mental health services.

Blackness is a significant factor in epistemic injustice however; it should not be considered in isolation from other intersecting identities. The structural concept of Whiteness in complex health systems is an equally important factor in identifying barriers to co-creative solutions that interacts with patient’s lived experiences of institutional racism in adapting for change for better public value outcomes for co-production to prosper [68]. Understanding has to be two-way, involving centring the lived experience of being White in parallel and in equal partnership of being Black [69].

Black men not wanting to be seen monolithically and defined by their (structurally pre-defined) racialised ethnic identity as ‘Black’ is not an attempt to minimise the impact, and effect of institutional racism. The wish to be de-racialised is constructed by Black men as an attempt to counter the existing negative Black societal stereotype of ‘big, Black and dangerous’ dominating psychiatry in wider ecologies in health care and British society at large [90,93]. Whilst anti-Blackness is the overshadowing reason, identified by Black men for poor mental health treatment in our study, there is a need to acknowledge that intra-communal differences do exist.

For example, Black men who belong to other marginalised groups, such as being gay or bisexual, face compounded stigma [93–96]. This intersectional stigma further affects their mental health and increases the risk of substance use, suicide and other psychosocial issue. There is, therefore, an importance to consider intersectional identities in mental health assessment and treatment and how this informs the need for tailored support whilst keeping anti-racist, anti-stigmatic practices at the forefront.

Addressing stigma is crucial to combat structural racism and institutional injustices [92,96]. This involves promoting mental health awareness and Black role models in the wider society, training professionals in handling trauma responses and PTSD presentations, and implementing policies that hold racist practices to account and ensure equitable access to care. The need for professionals to hear first-hand filmed accounts of the depth, effect and impact of generational, institutional racism on Black men is key to gaining empathy and a better understanding of detention experiences and improving professional’s responses.

Community based Interventions that focus on collaborations between social workers and community pillars, (e.g., barbers, faith leaders, etc) can create safe spaces for Black men to discuss mental health issues without fear of stigma or judgement. This can also help reduce negative mental health symptoms and prevent crisis. These partnerships can help dismantle negative stereotypes attached to Black men and mental health and promote mental well-being [69,80,97]. Suggestions of ‘how to’ are highlighted in our policy and practice recommendations.

## Strengths and limitations

The limitations of the EBCD adaptation, meant we did not capture idiographic accounts of Black men, nor were we able to explore detention experience in the depth we initially anticipated. This was due to new knowledge that emerged about to the lack of trust the local Black community had towards research/academia, the police, and mental health services/professionals (the direct stakeholder that straddle this project).

We initially felt the ‘failure’ to recruit for 1:1 interviews, could have weakened our study but after taking a step back, reflecting and changing our approach based on the community’s wishes it improved the engagement in a more authentic, and meaningful way with richer insights. This adaptation began to re-build a history of broken trust with the Black community research/academia, mental health and the police. Through the FGs people truly opened their heart when creating and reciting their poems or telling the story behind the masks they painted (and ‘wore’ all their lives). These became an unexpected strength of the project offered an authentic, meaningful data collection approach to re-build generations of mistrust.

Due to the stigma attached to mental health, FGs provided a safer place for men to speak openly about their experiences of racism, detention, mental health crisis, minimising any fear of judgement and gave light to the wider racial sociological complexities about mental health care. This demonstrates that if we only seek to improve services, we will only get better services however, humanising services includes the human interactions within services that prevent and/or heal a person’s trauma as well as treat the condition they entered services with. Therefore, what the team felt was an initial limitation became our study’s greatest strength.

## Conclusion

This study demonstrates that detention is not an isolated event but the beginning of a broader mental health system journey. Understanding and reforming this journey requires insights from Black men, their families, communities, and professionals to dismantle systemic racism and improve outcomes. This is the first study of its kind to unite professionals with Black lived experience stakeholders and report on their co-produced practice and policy recommendations.

In conclusion, the complexity of public health services requires ongoing adaptation and learning to achieve better outcomes. Smith [78] argues that the focus should shift from merely improving services to providing support, relationships, and understanding. This shift necessitates flexibility and experimentation in service delivery to address institutional and structural racism, which are significant barriers to co-creative solutions in mental health services. Institutional racism refers to systemic inequalities within psychiatric institutions, while structural racism encompasses broader societal practices that perpetuate racial inequalities. The study’s findings highlight the existence of structural racism within the mental health system, perpetuating institutional injustice for Black men and their communities. Implementing anti-discrimination laws, affirmative action, and data collection on racial disparities are crucial for achieving equity.

Public value in mental health involves co-creation and shared decision-making, where individuals with lived experiences engage in dynamic relationships to improve quality of life. Addressing epistemic injustice, where individuals’ knowledge is undervalued, requires open dialogue that values lived experiences as change agents to co-design practice, policy, and research. Stigma around mental health and the negative stereotyping of Black men exacerbate structural racism and institutional injustice. Addressing stigma involves promoting mental health awareness, trauma responsive approaches, training professionals, and implementing equitable policies. Community-based interventions can create safe spaces for Black men to discuss mental health issues, reducing stigma and promoting well-being. Implementing the study’s policy and practice recommendations is essential for achieving long-term change and improving mental health outcomes for Black men and their communities.
