# Correction: From mental health detention to health systems reform: Co-producing policy and practice recommendations with Black men, their communities, and health and social care professionals

**DOI:** 10.1371/journal.pmen.0000546

**Published:** 2026-01-27

**Authors:** Elaine Craig, Caroline Leah, Jeremy Dixon, Anna Bergqvist, Isaiah Brodrick, Debbie Best, Kim Heyes, Joy Duxbury, Alina Haines-Delmont

The affiliation for the fourth and sixth author is incorrect. Anna Bergqvist and Debbie Best are not affiliated with #4 but with #1: School of Nursing and Public Health, Manchester Metropolitan University, United Kingdom.
